# Reduction in asthma deteriorations in subjects with persistent asthma uncontrolled on low-, medium-, or high-dose inhaled corticosteroids: a pooled analysis from three clinical trials using combined mometasone furoate/formoterol

**DOI:** 10.1186/1710-1492-7-S2-A13

**Published:** 2011-11-14

**Authors:** SF Weinstein, RA Nathan, EO Meltzer, D Gates, H Nolte

**Affiliations:** 1 Allergy and Asthma Specialists Medical Group and Research Center, Huntington Beach, CA, USA; 2 Asthma & Allergy Associates, P.C. and Research Center, Colorado Springs, CO, USA; 3 Allergy and Asthma Medical Group and Research Center, San Diego, CA, USA; 4 Merck Research Laboratories, Kenilworth, NJ, USA

## Objective

We present a post hoc analysis from 3 phase III clinical trials examining the effects of mometasone furoate/ formoterol (MF/F) combination therapy on asthma deterioration (ie, severe asthma exacerbation) in subjects previously uncontrolled on low-, medium-, or high-dose inhaled corticosteroids (ICS).

## Methods

A 2–3-week run-in period with twice daily (BID) MF-100µg, MF-200µg, or MF-400µg was performed before subject (≥12y) randomization to BID: MF/F-100/10µg, MF-100µg, F-10µg, or placebo for 26weeks (n=746); MF/F-200/10mg, MF-200µg, F-10µg, or placebo for 26weeks (n=781); MF/F-200/10µg, MF/F-400/10µg, or MF-400µg for 12weeks (n=728 ). Assessment of asthma deterioration (ie, 20% decrease in forced expiratory volume in 1s [FEV_1_]; 30% decrease in peak expiratory flow [PEF] on ≥2 consecutive days; or clinically judged deterioration [ie, emergency treatment, hospitalization, or treatment with excluded medications]) was a co-primary endpoint for studies, and a secondary endpoint for study. Post hoc pair-wise comparisons of pooled MF/F vs MF, F, and placebo treatment groups were performed.

## Results

Sample sizes in this pooled analysis were: MF/F, n=861; MF, n=620; F, n=390; placebo, n=384. There was a significantly lower incidence of asthma deterioration with MF/F= 17.2% vs MF=26.1% (*P*=.002), F=49.5% (*P*<.001), and placebo550.8% (*P*<.001). Incidence rates for asthma deterioration subtypes were: FEV_1_ reduction: MF/F=7.0%, MF=10.0%, F=13.8%, placebo=17.7%; PEF reduction: MF/F=7.5%, MF=12.6%, F=27.2%, placebo=26.3%; clinically judged deterioration: MF/F=2.1%, MF=2.6%, F=6.7%, placebo=5.2%.

## Conclusion

MF/F-treated subjects experienced a significantly lower rate of asthma deterioration compared with MF, F, and placebo in subjects previously uncontrolled on low-, medium-, or high-dose ICS in this pooled analysis.

